# Cortical neurons are a prominent source of the proinflammatory cytokine osteopontin in HIV-associated neurocognitive disorders

**DOI:** 10.1007/s13365-015-0317-3

**Published:** 2015-01-31

**Authors:** Katie Silva, Calixto Hope-Lucas, Tyesha White, Tai-Kyung Hairston, Tatenda Rameau, Amanda Brown

**Affiliations:** Department of Neurology, Johns Hopkins University School of Medicine, 600 North Wolfe Street/Meyer 6-181, Baltimore, MD 21287-7131 USA

**Keywords:** Neuroinflammation, Neurodegeneration, CD68, Iba-1, AIF-1, Inflammation, Macrophage, Microglia, Astrocytes, Systemic inflammation

## Abstract

The proinflammatory cytokine osteopontin (OPN) is elevated in the cerebrospinal fluid (CSF) in individuals with HIV-associated neurocognitive disorders (HAND) and remains so in those on suppressive antiretroviral therapy. To understand the pathophysiological significance of elevated OPN in the CNS, we sought to determine the cellular source of this cytokine. As HIV-1 replicates productively in macrophages/microglia, we tested whether these cells are the predominant producers of OPN in the brain. Stringent patient selection criteria, which excluded brain tissues from those with evidence of drug abuse and dependence, were used. Uninfected normal controls, amyotrophic lateral sclerosis (ALS), HIV+ asymptomatic neurocognitive impairment (ANI), and HIV+ mild neurocognitive disorder (MND)/HIV-associated dementia (HAD) groups were included. Double-label immunohistochemistry for CNS cells and OPN was used to quantify OPN expression in astrocytes, macrophages/microglia, and neurons. While resident macrophages/microglia expressed OPN, astrocytes and unexpectedly neurons were also a major source of OPN. OPN levels in ionized Ca^2+^-binding adapter 1 (Iba1)/allograft inflammatory factor-1 (AIF-1)+ microglia in HIV+ ANI and MND/HAD exceeded those of HIV-negative controls and were comparable to expression seen in ALS. Moreover, in neurons, OPN was expressed at the highest levels in the HIV+ ANI group. These findings suggest that while infiltrating HIV-infected macrophages are most likely the initial source of OPN, resident CNS cells become activated and also express this inflammatory cytokine at significant levels. Moreover, as OPN levels are elevated compared to uninfected individuals and increases with the severity of impairment, it appears that the expression of OPN is persistent and sustained within the brain parenchyma in those that progress to HAND.

## Introduction

Trafficking of HIV-infected macrophages and activated uninfected monocytes/macrophages into the brain occurs early in the course of viral infection (Budka et al. 1987; Davis et al. 1992; Glass et al. 1995; Koenig et al. 1986) and initiates the seeding of resident brain macrophages, microglia, and astrocytes with HIV/SIV DNA (Clements et al. 2002). The local immune response in the brain results in an inflammatory milieu that exacerbates the release of substances including glutamate leading, by mechanisms that remain to be fully elucidated, to neurotoxicity, neuronal dysfunction, and cognitive impairment collectively known as HIV-associated neurocognitive disorders (HAND) (Kraft-Terry et al. 2010; Vázquez-Santiago et al. 2014). HAND is assessed through a battery of neuropsychological tests and encompasses three levels of impairment: asymptomatic neurocognitive impairment (ANI), HIV-associated mild neurocognitive disorder (MND), and HIV-associated dementia (HAD) (Antinori et al. 2007). The prevalence of HIV-associated neurocognitive disorders and particularly the milder forms has increased, while the incidence of severe debilitating dementia has dramatically decreased (McArthur et al. 2010). This latter finding reflects the high efficacy of suppressing HIV replication, which results in significant reductions in viral load in the blood and cerebrospinal fluid (CSF) with combination antiretroviral therapy (ART) that target two or more steps in the viral life cycle. However, in up to 50 % of HIV-infected individuals on ART, cognitive dysfunction remains a significant problem (Heaton et al. 2010; Heaton et al. 2011), and currently, no adjunctive neuroprotective therapies have yet reached routine clinic practice (Meulendyke et al. 2014).

Several potential explanations have been proposed to explain the continued impairment including the inefficient penetration of ART into the brain parenchyma that would permit low-level ongoing viral replication at this site, toxicity of ART and its metabolites (Tovar-y-Romo et al. 2012), and comorbidities including aging and cardiovascular disease could all potentially contribute to the continued dysfunction seen in HIV-infected individuals (Deeks et al. 2013). Additionally, markers of immune activation remain elevated in the periphery and CNS in even the most well-suppressed HIV-infected ART-treated individuals, suggesting the continued stimulation and hyperactivity of the immune system to antigens from several anatomical locations including the intestine (Brenchley et al. 2006; Spudich et al. 2011). Inflammation in the brain in response to injury or invasion by microorganisms can play either beneficial and/or neuropathogenic roles depending on the intensity and duration of the response (Katsumoto et al. 2014).

Osteopontin (OPN), a proinflammatory cytokine of the innate immune system, was first reported to be increased in the brains of SIVE-infected macaques and later in the plasma, CSF, and brains of HIV-infected individuals with cognitive impairment (Brown et al. 2011; Burdo et al. 2008). A recent study suggests that OPN remains elevated in plasma in those on ART (Burdo et al. 2011). Interestingly, OPN is elevated in other neurodegenerative disorders including multiple sclerosis (MS), Alzheimer’s disease, Parkinson’s disease, and frontotemporal dementia (Braitch et al. 2008; Chabas et al. 2001; Comabella et al. 2005; Comi et al. 2010; Iczkiewicz et al. 2006; Maetzler et al. 2007; Mattsson et al. 2008; Wirths et al. 2010). Only in the case of MS has the role of OPN been explored where in mouse models of the disorder, OPN has been shown to facilitate the survival of anti-myelin autoreactive T cells (Hur et al. 2006; Jansson et al. 2002). We have shown that OPN is significantly elevated in HIV-infected macrophages (Brown et al. 2011). Inhibition of OPN in macrophages with siRNA inhibits HIV replication 50 % suggesting that OPN stimulates viral replication likely through a NF-κB-dependent mechanism acting via OPN receptors, thus forming a feedback loop which promotes continued cellular activation and viral spread (Brown et al. 2011; Eger et al. 2014). Because of their central role in the development of HIV-associated cognitive impairment, and to gain an understanding of the impact of activated HIV-infected macrophages on the expression of OPN in the context of the central nervous system (CNS), we used double-label immunochemistry for cells of the human brain and OPN to test the hypothesis that resident macrophages/microglia are the major sources of this proinflammatory cytokine associated with HAND. Four groups of brain tissue were compared including normal, HIV-uninfected controls, HIV-positive with ANI, HIV-positive MND/HAD, and a group with amyotrophic lateral sclerosis (ALS). ALS is a progressive motor neuron degenerative disorder, in which macrophage and microglial activation is now thought to be involved in the pathological progression of the disease. Moreover, recent studies suggest that microglial activation may be associated with deficits in executive function (Brettschneider et al. 2012a; Brites and Vaz 2014). As such, ALS samples were included as a “neurodegenerative other” control. While astrocytes and resident macrophage/microglia express OPN, to our surprise we found that in addition, neurons significantly contribute to the pool of OPN found in the brain in individuals with HAND. These findings suggest that while HIV-infected macrophages stimulate the initial increase of OPN in the brain, resident macrophage/microglia, astrocytes, as well as neurons overexpress this cytokine. As all of these cells express the receptors for OPN (Ailane et al. 2013; Akiyama et al. 1993), our findings suggest that microglial, astrocytic, and neuronal activity and function can potentially be directly altered by OPN through paracrine regulatory mechanisms as a result.

## Results

### Patient samples and clinical characteristics

Microarray analyses on the brains of SIV-infected macaques identified numerous genes including osteopontin (OPN) that were differentially regulated (Roberts et al. 2003). Further investigations found that OPN was significantly elevated in the plasma of rhesus macaques and CSF and brain in individuals with HAND (Brown et al. 2011; Burdo et al. 2008; Eger et al. 2014). Burdo et al. proposed HIV-infected and/or activated macrophage trafficking into the brain releases OPN, which has chemotactic and antiapoptotic signaling potential, and serves in a feedback loop fueling the establishment of HIV in the brain as well as the cellular activation and inflammation at this site (Burdo et al. 2007). To better understand the potential neuropathogenic role for elevated OPN in HAND, in this study, we tested the hypothesis that activated macrophages/resident microglia are the cellular sources of OPN in the brain. Stringent patient sample criteria were used including the exclusion of the drugs of abuse cocaine, cannabis, opiate, and methadone. In addition, toxicology reports were examined to exclude drug abuse and dependence. We used double-label immunohistochemistry and antibodies to CD68 and Iba-1/AIF-1 to specifically identify macrophage/microglia in brain tissue from uninfected controls, HIV+ ANI, HIV+ MND/HAD, and a group with a well-known neurodegenerative disease affecting motor neurons, amyotrophic lateral sclerosis, ALS. Moreover, as astrocytes and neurons have been reported to express increased levels of OPN in models of brain injury and neurodegeneration, we also used anti-glial fibrillary acidic protein (GFAP) and anti-neuronal nuclei (NeuN) antibodies to determine whether this proinflammatory cytokine is elevated in these cell types. The demographics and clinical parameters of the patients used are shown in Table 1. There were no significant differences in age of the subjects in each group (normal, 47.7 ± 7.35; ALS, 50.4 ± 7.37, HIV ANI 43.4 ± 7.89; HIV NMD/HAD, 40.5 ± 10.5).

**Table 1:** Patient sample demographics

Patient ID	Age	Sex	Race	HIV status	Neurocog diagnosis	Plasma VL	CSF VL	CD4	ARV
Case 1	47	F	Black	Neg	Normal	NA	NA	NA	NA
Case 2	44	F	White	Neg	Normal	NA	NA	NA	NA
Case 3	51	M	White/Hispanic	Neg	Normal	NA	NA	NA	NA
Case 4	49	M	White	Neg	Normal	NA	NA	NA	NA
Case 5	46	M	White	Neg	Normal	NA	NA	NA	NA
Case 6	39	M	Hispanic	Pos	ANI	570	<50	79	Yes
Case 7	43	M	White	Pos	ANI	249	19	7	Yes
Case 8	39	M	White	Pos	MND	72,125	70	1	Yes
Case 9	33	M	Hispanic	Pos	MND	400	NT	11	Yes
Case 10	35	M	White	Pos	MND/HAD (HIVE)	2827	78	211	Yes
Case 11	37	M	White	Pos	MND	178	5968	85	Yes
Case 12	31	M	White	Pos	MND	483,758	148	10	Yes
Case 13	57	M	Asian	Pos	HAD	40,133	2747	299	Yes
Case 14	30	F	Hispanic	Neg	Normal	NA	NA	NA	NA
Case 15	48	F	Hispanic	Neg	Normal	NA	NA	NA	NA
Case 16	50	M	Hispanic	Neg	Normal	NA	NA	NA	NA
Case 17	56	F	White	Neg	NA	NA	NA	NA	NA
Case 18	57	M	Black	Pos	MND	63,953	NT	194	Yes
Case 19	34	M	White	Pos	ANI	>750,000	NT	9	Yes
Case 20	46	M	White	Pos	ANI	<50	<50	233	Yes
Case 21	45	M	Hispanic	Pos	MND/HAD	628	376	28	Yes
Case 22	31	F	Hispanic	Pos	MND/HAD	744,349	NT	4	Yes
Case 23	55	F	Black	Pos	ANI	37,060	NT	114	Yes
Case 24	56	F	White	Neg	Normal	NA	NA	NA	NA
Case 25	38	M	White	Neg	ALS	NA	NA	NA	NA
Case 26	53	F	White	Neg	ALS	NA	NA	NA	NA
Case 27	50	M	White	Neg	ALS	NA	NA	NA	NA
Case 28	54	M	White	Neg	ALS	NA	NA	NA	NA
Case 29	57	F	Hispanic	Neg	ALS	NA	NA	NA	NA

Data are from last clinic visit. Age at death

*NA* not applicable, *NT* not available, *HIV Neg* seronegative, *HIV Pos* seropositive, *VL* viral load, *ARV* antiretroviral use, *ANI* asymptomatic neurocognitive impairment, *MND* minor neurocognitive disorder, *HAD* HIV-associated dementia, *ALS* amyotrophic lateral sclerosis

### Osteopontin is significantly elevated in GFAP-reactive astrocytes from HIV+ MND/HAD cases compared to ALS samples

**Fig. 1:**
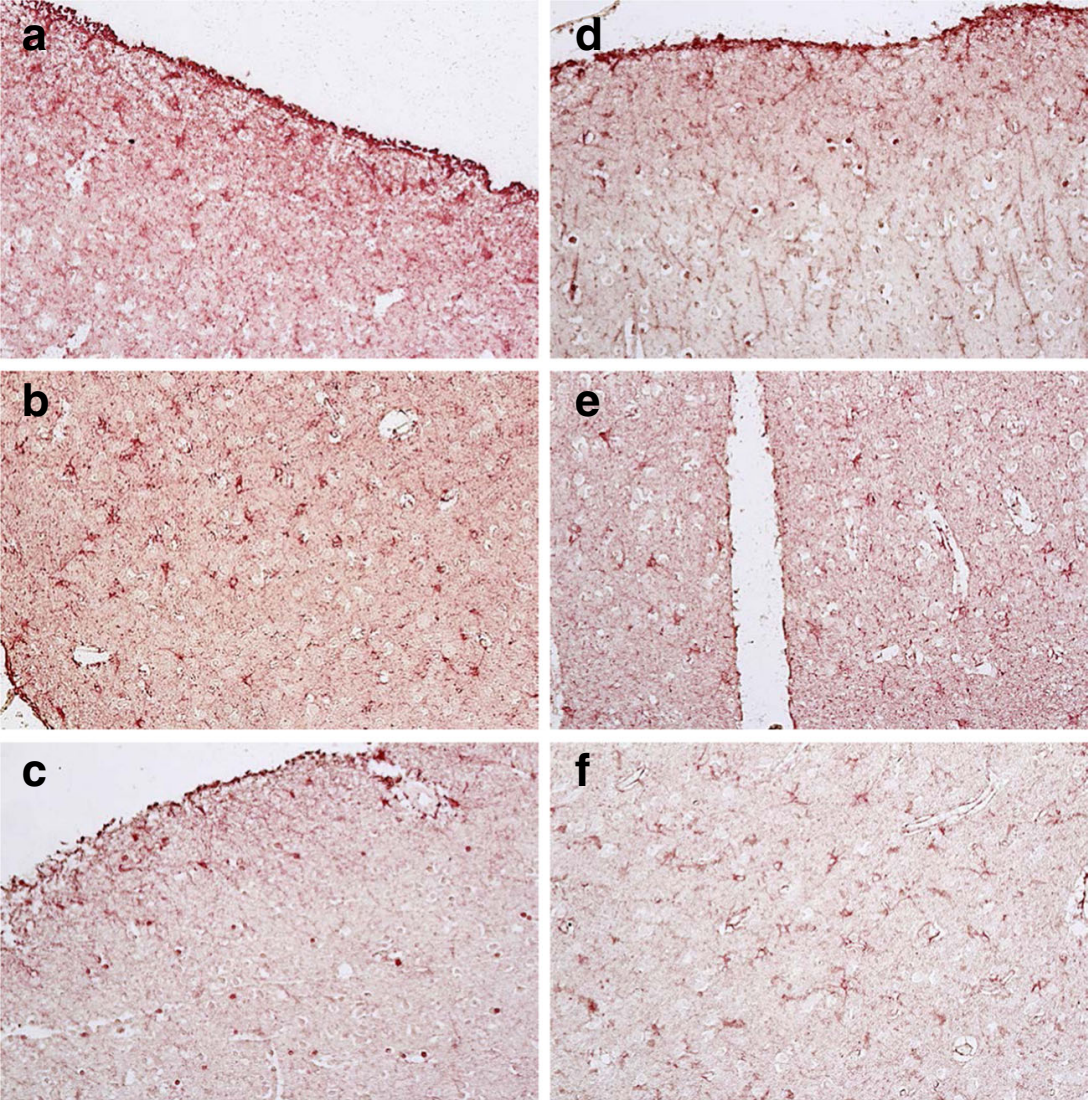
Osteopontin (OPN) expression in GFAP+ astrocytes. Paraffin-embedded human autopsy tissue from the occipital lobe of cases and controls from the National NeuroAIDS Tissue Consortium (NNTC) was stained sequentially with rabbit polyclonal antisera against GFAP/goat-anti-rabbit-alkaline phosphatase (AP) secondary and developed with permanent Fast-Red Quanto (*red*) then reacted with mouse monoclonal antibody to OPN/goat anti-mouse-horse radish peroxidase and developed with 3,3′-diaminobenzidine. **a** case 14, normal control; **b** case 29, amyotrophic lateral sclerosis (ALS); **c** case 19, asymptomatic neurocognitive disorder (ANI); **d** case 20, ANI; **e** case 21, minor neurocognitive disorder/HIV-associated dementia (MND/HAD); **f** case 22, MND/HAD

**Fig. 2:**
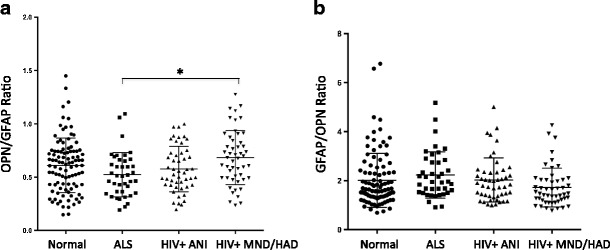
Osteopontin is significantly elevated in GFAP-reactive astrocytes from HIV+ MND/HAD cases compared to amyotrophic lateral sclerosis (ALS) samples. Ten images were taken at ×20 magnification from all four quadrants of the slides for each case (an equal amount of area), and the intensity (area fraction) of the label for OPN and GFAP were quantified using ImageJ. The results are expressed as the ratio of **a** OPN to GFAP or **b** GFAP to OPN. One-way ANOVA, with Tukey’s correction for multiple comparisons and significance of *p < 0.05, was determined with GraphPad Prism 6. Normal, ALS, amyotrophic lateral sclerosis, HIV-infected cases asymptomatic neurocognitive disorder (HIV+ ANI), and HIV-infected cases with minor neurocognitive disorder/HIV-associated dementia (HIV+ MND/HAD)

GFAP reactivity was highest in the upper cortical layers in most samples and in some cases, positively stained cells were visible throughout all cortical layers (Fig. 1). In the HIV+ MND/HAD group, astrocytic cell bodies devoid of processes often predominated (Fig. 1, cases 19 and 20). Compared to the normal control group (0.612 ± 0.255, *n* = 9), OPN levels did not differ significantly with the other groups (HIV + NC, 0.576 ± 0.212, *n* = 5; HIV+ impaired, 0.682 ± 0.254, *n* = 5; ALS, 0.524 ± 0.207, *n* = 5), although there was a trend of increased OPN in the HIV+ MND/HAD group (Fig. 2a). A significant increase in the level of OPN between the HIV+ MND/HAD and the ALS group was detected (*p* = 0.011, Fig. 2). Normalization of GFAP levels to OPN revealed no significant differences in the expression of GFAP among the different groups (Fig. 2b; normal, 2.02 ± 1.10; HIV + ANI, 2.027 ± 0.898; HIV+ MND/HAD 1.722 ± 0.792; ALS, 2.231 ± 0.948). These results suggest that GFAP-reactive astrocytes in HAND express OPN, and while levels between HIV+ NC and HIV+ MND/HAD did not differ, astrocytes in the latter case produce significantly higher levels of OPN than what was seen in samples from individuals with ALS.

### Microglial osteopontin levels are significantly increased in HAND and ALS

**Fig. 3:**
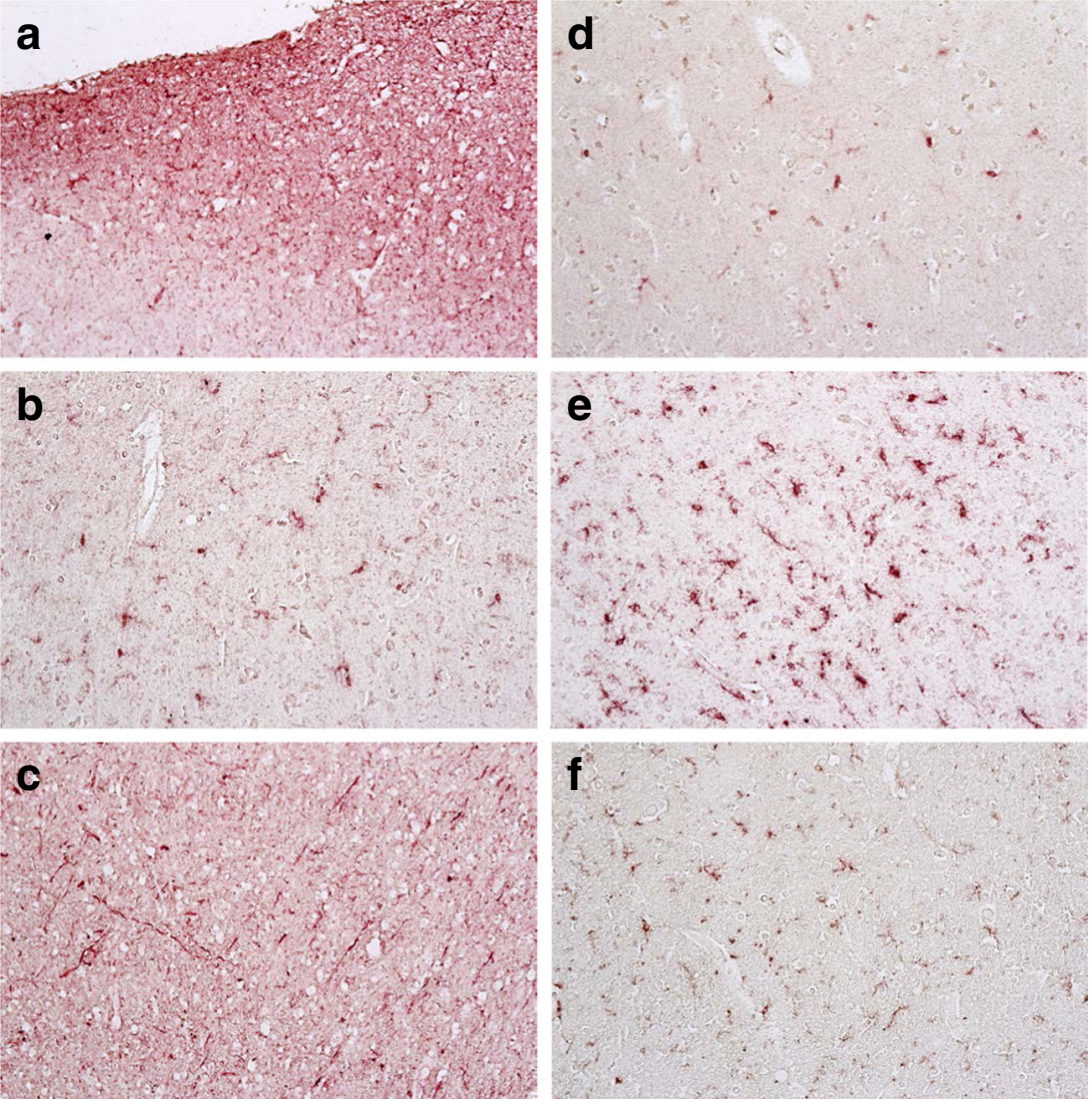
Osteopontin (OPN) expression in Iba1/AIF-1+ microglia. See staining paradigm as given in figure legend 1. **a** case 16, normal; **b** case 4, normal; **c** case 29, ALS; **d** case 23, ANI; **e** case 12, MND; **f** case 13, HAD

**Fig. 4:**
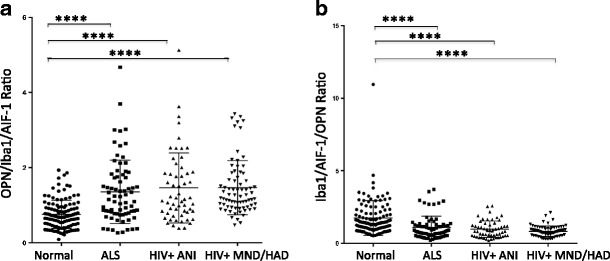
Microglia-associated osteopontin levels are significantly increased in HAND and ALS. See description of quantification scheme given in figure legend 2. The results are expressed as the ratio of **a** OPN to Iba1/AIF-1 or **b** Iba1/AIF-1 to OPN. One-way ANOVA, with Tukey’s correction for multiple comparisons and significance of *p* < 0.05, was determined with GraphPad Prism 6. Normal, ALS, amyotrophic lateral sclerosis, HIV-infected cases asymptomatic neurocognitive disorder (HIV+ ANI), and HIV-infected cases with minor neurocognitive disorder/HIV-associated dementia (HIV+ MND/HAD); ****p < 0.0001

Microglia stained for Iba-1/AIF-1 displayed a variety of morphologies including ramified and ameboid shapes with varying degrees of processes (Fig. 3). Unexpectedly, abundant Iba-/AIF-1 staining was seen in several samples from the normal control group (Fig. 3a–b, cases 16 and 4). In addition, there was abundant Iba-1/AIF-1 reactivity in samples from the ALS group with distinctive labeling of microglia with extended ramified shapes (Fig. 3c, case 29). OPN levels in microglia were significantly elevated compared to normal controls (0.758 ± 0.376, *n* = 9) for ALS (1.356 ± 0.848, *p* < 0.0001, *n* = 5), HIV+ ANI (1.463 ± 0.934, *p* < 0.0001, *n* = 5), and HIV+ MND/HAD (1.467 ± 0.721, *p* < 0.0001, *n* = 5), but there were no differences between the disease groups (Fig. 4a). The level of Iba-I/AIF-1 normalized to OPN intensity was significantly lower in the disease groups (HIV+ ANI, 0.969 ± 0.570, *p* < 0.0001; HIV+ MND/HAD, 0.829 ± 0.358, *p* < 0.0001; ALS, 1.094 ± 0.782, *p* < 0.0001) compared to the normal controls (1.741 ± 1.21) (Fig. 4b). These results suggest that the amount of OPN expression per microglia was significantly elevated in the diseased groups compared to the normal controls.

### Osteopontin levels in cortical neurons are significantly elevated in HIV-infected individuals with ANI compared to HIV-infected cases with cognitive impairment (MND/HAD) or ALS

**Fig. 5:**
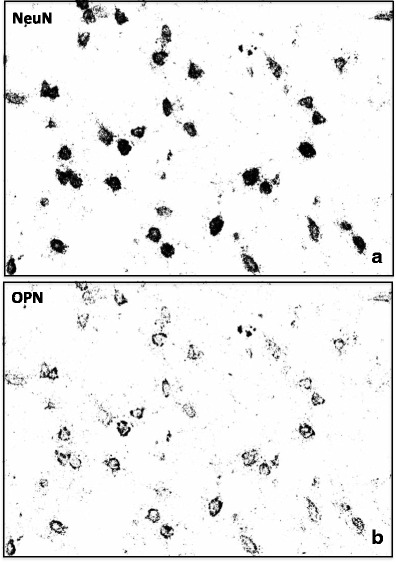
NeuN reactivity is localized to the nucleus and cytoplasm, while OPN is restricted to the cytoplasm of neuronal cell bodies. Image label intensity for NeuN and OPN reactivity in double-stained sections were analyzed using ImageJ. *Top panel*, threshold for NeuN positivity. *Lower panel*, threshold for anti-OPN staining within neurons

**Fig. 6:**
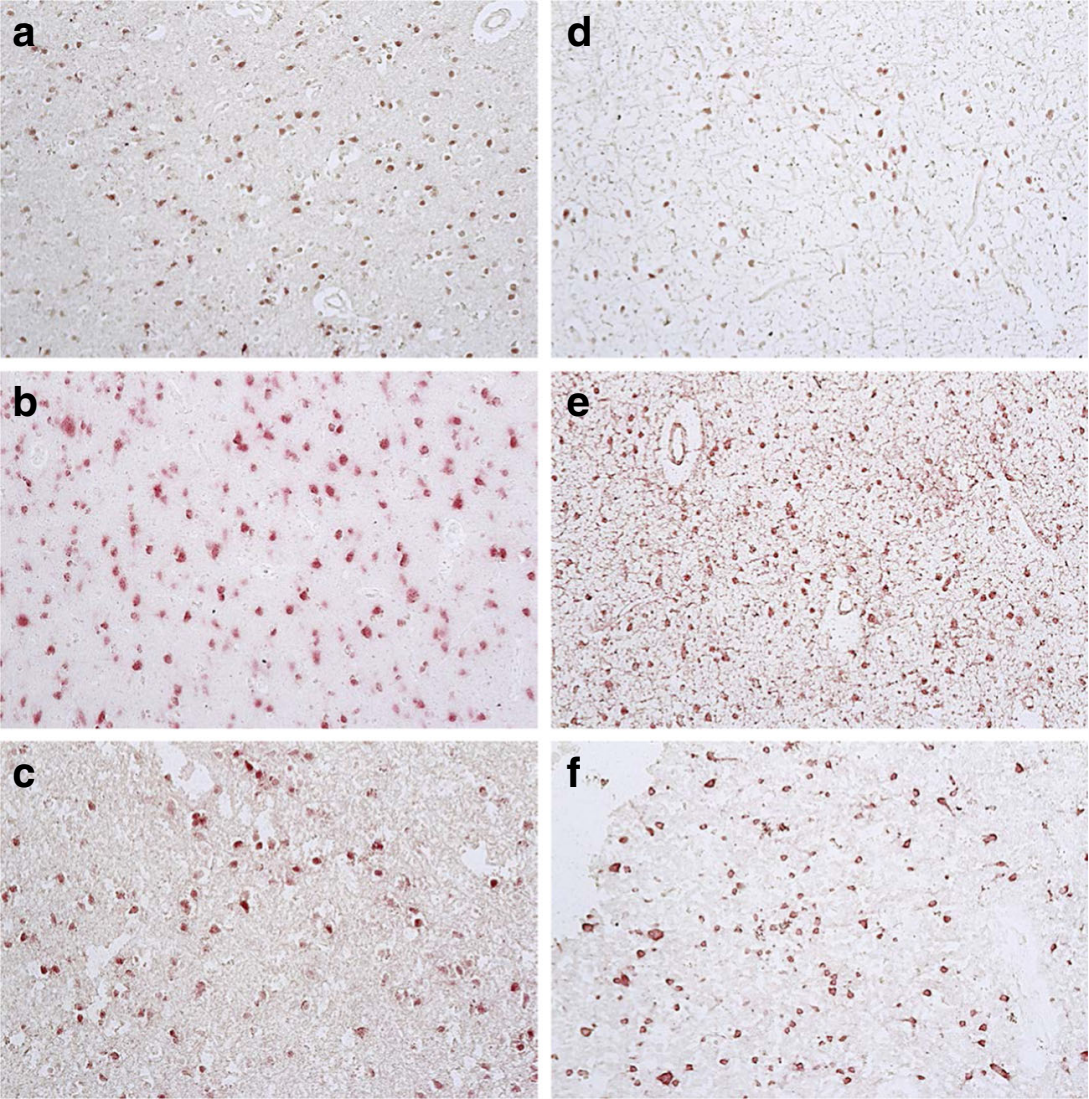
Double-labeled neurons expressing NeuN and OPN. See staining paradigm as given in figure legend 1. **a** case 16, normal; **b** case 25, ALS; **c** case 18, MND; **d** case 20, ANI; **e** case 21, MND/HAD; **f** case 13, HAD

**Fig. 7:**
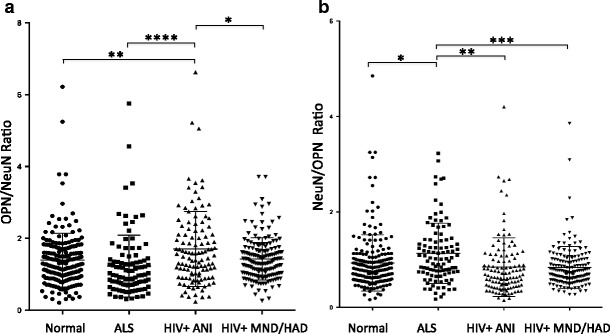
Osteopontin levels in cortical neurons are significantly elevated in HIV-infected individuals with asymptomatic neurocognitive impairment (ANI) compared to HIV-infected cases with cognitive impairment (MND/HAD) or ALS. See description of quantification scheme given in figure legend 2. The results are expressed as the ratio of **a** OPN to NeuN, ****p < 0.0001, **p = 0.0052, *p = 0.0232, or **b** NeuN to OPN, ****p < 0.0001, ***p = 0.0002, **p = 0.0011, *p = 0.0181. Normal, ALS, amyotrophic lateral sclerosis, HIV-infected cases asymptomatic neurocognitive disorder (HIV+ ANI), and HIV-infected cases with minor neurocognitive disorder/HIV-associated dementia (HIV+ MND/HAD)

In HIV+ and ALS samples, NeuN reactivity was localized in the nucleus as well as in the cytoplasm (Lucas et al. 2014), while OPN staining was restricted to the cytoplasm of neuronal cell bodies (Fig. 5). Unexpectedly, abundant OPN reactive NeuN+ neurons were readily detectable in many of the normal uninfected controls (Fig. 6a). OPN levels were highest in the HIV+ ANI cases (1.706 ± 1.045, *n* = 6) compared to all other groups (normal, 1.394 ± 0.748, *p* = 0.005, *n* = 10; HIV+ MND/HAD, 1.428 ± 0.589, *p* = 0.023, *n* = 8; ALS, 1.226 ± 0.862, *p* < 0.0001, *n* = 5) (Fig. 7). Based on the NeuN/OPN ratio, the ALS group (1.134 ± 0.632) had significantly more NeuN-reactive neurons compared to the normal (0.93 ± 0.59, *p* = 0.018), HIV+ ANI (0.844 ± 0.614, *p* = 0.001), and HIV+ MND/HAD groups (0.836 ± 0.438, *p* = 0.0002), which were similar to each other (Fig. 7b). These results suggest that neurons in cases from HIV+ ANI produce, on average, more OPN than all other groups.

### CD68+ macrophages/microglia are readily detectable in normal control samples

**Fig. 8:**
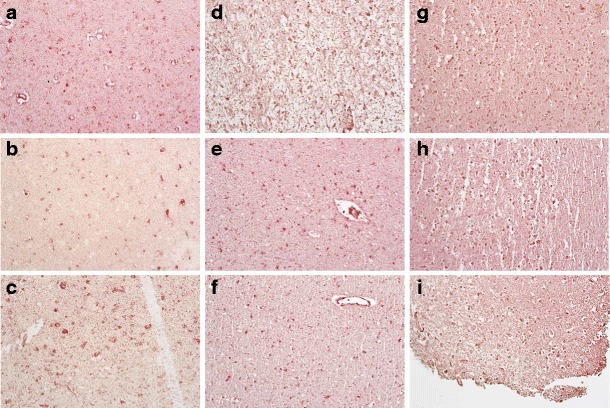
Presence of CD68+ macrophages/microglia in normal and MND/HAD groups. See staining paradigm as given in figure legend 1. **a** case 14, normal; **b** case 24, normal; **c** case 16, normal; **d** case 1, normal; **e** case 17, normal; **f** case 5, normal; **g** case 2, normal; **h** case 11, MND; and **i** case 13, HAD

**Fig. 9:**
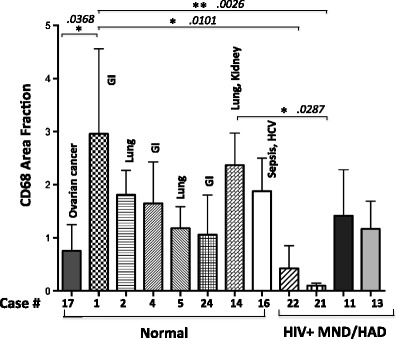
CD68+ macrophages/microglia are readily detectable in normal control samples. ImageJ was used to quantify the area fraction of CD68 reactivity in three representative fields from each case. The mean and standard deviation and level of significance as determined by one-way ANOVA for normal controls and MND/HAD cases are shown. Category of clinical disorders (Table 2) at the time of death is indicated *above the appropriate bars*: *GI* gastrointestinal related, *HCV* hepatitis C virus

The high level of OPN, GFAP, and Iba-1/AIF-1 expression seen in the normal control group suggested the presence of injury and/or inflammation in the brain. To investigate this possibility, we stained sections with anti-CD68, which detects microglia, as well as perivascular, parenchymal, and resident macrophages (Esiri and McGee 1986), which typically have a finite lifespan in the brain and are not present in large numbers under normal homeostatic conditions (Esiri and O’D McGee 1986; Fischer-Smith et al. 2004). Strikingly, in 8 out of 10 normal cases, CD68+ macrophages/microglia were readily detectable throughout the cortical layers (Fig. 8). The level of CD68 reactivity was similar to or significantly exceeded that of HIV+ MND/HAD samples (case 1 vs. case 21, *p* = 0.0026; case 1 vs. case 22, *p* = 0.0101; case 14 vs. case 21, *p* = 0.0287; Fig. 9). Table 2 lists the cause of death for samples in the normal control group. In several cases, severe injury to the gastrointestinal tract (Fig. 9, cases 1, 4, and 24) and disorders of the lungs (Fig. 9, cases 2, 5, and 14) displayed the highest levels of CD68 staining, although there were no statistically significant differences between the normal samples with the exception of case 17 vs. case 1 (*p* = 0.0368). These results suggest that the majority of normal controls used in this study, although not infected with HIV, displayed evidence of exposure to an event(s) that promoted a sustained increase of CD68+ macrophages in the brain.

**Table 2:** Clinical indications at the time of death

Patient ID	Disorders at time of death
Case 1	Upper GI bleed from ruptured esophageal varices; autoimmune hepatitis, ANA+ arthritis
Case 2	Tension pneumothorax most likely due to puncture of lung
Case 3	Multi-organ failure secondary to cardiogenic shock due to myocardial infarction in a failing heart with prior ischemic damage
Case 4	Hemorrhagic shock due to gastrointestinal bleeding from a large duodenal ulcer that destroyed a segment of the tastroduodenal artery; peptic ulcer disease
Case 5	Severe necrotizing tracheobronchitis and pneumonia due to community acquired-MRSA and beta hemolytic Streptococcus group B
Case 14	Severe mitral valvular disease with calcified bioprosthesis and calcific, infected emboli to multiple organs (consistent with strep pneumoniae); end-stage renal disease; bronchopneumonia
Case 15	Hepatitis C cirrhosis and end stage liver disease; staph aureus sepsis; prior IVDU
Case 16	Klebsiella sepsis with hepatic abscess, OLT for HCV cirrhosis, pulmonary hypertension, ECRP with sphincterotomy for biliary stones
Case 24	Scleroderma with CREST syndrome, GI hypomotility with large bowel obstruction, subtotal colectomy, erosive candidal esophagitis, and pneumoatosis intestinalis small intestine; pulmonary fibrosis, hypertension, and emphysema; arterionephrosclerosis; cachexia
Case 17	Ovarian cancer

## Discussion

The high level of OPN staining in GFAP+ astrocytes and NeuN+ neurons in the HIV-uninfected control group was very surprising. For these types of studies, normal controls present several limitations: they are less plentiful and more difficult to obtain, and specific clinical data most relevant to the study is often not available. The presence of a high concentration of CD68+ macrophage/microglia strongly suggests that these brains experienced injury and/or pathogenic sequela, which resulted in an inflammatory response. In fact, there is now greater recognition and study of the relationships between injury in the kidneys, lungs, and gastrointestinal tract and inflammation in the central nervous system (Bémeur and Butterworth 2013; Hilzendeger et al. 2014; Winterberg and Lu 2012). While all attempts are made to select the most appropriate controls, there are inherent limitations when working with human clinical samples. Perhaps prescreening of normal controls for specific inflammatory markers could be used to select tissues with low levels of baseline activation. It should be noted that the samples in this study differed from those used in a prior report on OPN levels in the CSF (Brown et al. 2011).

Despite the possible limitations of the control samples used in this study, OPN was expressed at a significantly higher level in Iba-1/AIF-1+ microglia in both the HIV+ ANI and HIV+ MND/HAD groups suggesting that these cells are a major source of this proinflammatory cytokine in the brain. The HIV+ samples came from individuals who were on ART, yet elevated OPN levels appeared to be sustained over a period of many years. A recent study analyzing traditional markers of cellular activation using brain tissue from the frontal white matter and basal ganglia of HIV-infected individuals on ART with cognitive impairment, but no encephalitis, also found that inflammation persists in those without HIV encephalitis despite therapy (Tavazzi et al. 2014). Elevation of CD68+ macrophage/microglia in the absence of any evidence of local proliferation in autopsy samples from the hippocampus of individuals on highly active antiretroviral therapy compared to normal controls was seen in a study by Anthony et al. suggesting that these cells remain activated despite the effective suppression of HIV replication (Anthony et al. 2005). However, unlike our study, many of the samples were from individuals who abused drugs (and were HCV+), a potential contributor to neuroinflammation (Anthony et al. 2005). Moreover, in the current study, 11 out of the 14 HIV-infected individuals, irrespective of detectable viral load in the plasma or CSF, were very immunosuppressed as determined by a CD4+ T cell level below 200 cells/ml (Table 1). In 50 % of the HIV+ cases, viral load was detected in the CSF suggesting the real possibility that persistent viral replication albeit at varying levels continued within the brain parenchyma.

Ionized Ca^2+^-binding adapter 1 (Iba1), which is thought to be the same gene product as allograft inflammatory factor-1 (AIF-1), is a 17-kDa cytoplasmic protein that binds calcium whose function continues to be elucidated (Zhao et al. 2013). While Iba-1/AIF-1 is expressed constitutively, it can also be a marker of the cellular inflammatory response as it sits in a gene cluster with TNF-α, TNF-β, and NF-κB in the MHC class III region (Zhao et al. 2013). Both ameboid and ramified microglia morphology was observed in most cases. Both types of microglia possess phagocytic activity and the ability to clear debris, while those with a ramified shape have been reported to contribute to efficient neurogenesis and the maintenance of synapses (Paolicelli et al. 2014; Perry and Teeling 2013). In the HIV+ MND/HAD group, microglia without any processes, which may be the ameboid type, often predominated suggesting that they are actively involved in phagocytosis and the clearance of dying cells. Alternatively, the absence of microglia with ramified morphology could indicate a functional deficit in the ability to conduct the high-level surveillance that is characteristic of microglia in the unimpaired brain (Nimmerjahn et al. 2005). In this regard, analyses of the Iba-1/OPN ratio showed that Iba-1 levels were significantly lower in all of the disease groups compared to the control.

This study is the first to show that OPN is also significantly elevated in activated microglia in ALS. The rod-like shape of microglia seen in this study has been recently reported in a rat model of brain injury (Ziebell et al. 2012) and was seen earlier in a model of experimental autoimmune encephalomyelitis or neuritis (Schluesener et al. 1998). Additional studies with a larger sample set and at different disease stages are required to determine whether the increase in OPN displays a temporal pattern. Interestingly, a recent study found that CD44, the receptor for OPN, is elevated in the SOD1 mouse model for familial ALS (Matsumoto et al. 2012). A connection between disease progression, deficits in executive function, and high levels of microglial activation has been found in ALS and is of relevance as this area of cognitive functioning is also compromised in HIV-infected individuals (Brettschneider et al. 2012a; Brettschneider et al. 2012b)

Our data revealed that in addition to microglia, neurons in HAND are a major source of OPN. Levels of OPN were highest in the HIV+ ANI group. This difference was not related to the overall levels of NeuN, as they were similar in the HIV+ ANI and HIV+ MND/HAD groups. A possible limitation is the small sample size as outliers could skew the results. In a prior study, we found that OPN levels in CSF were elevated in HIV-infected individuals with normal cognition and further increased with a higher level of impairment (Brown et al. 2011). Increases in OPN in the CSF, and specifically in neurons, have been reported in neurodegenerative disorders including multiple sclerosis, Alzheimer’s and Parkinson’s diseases, frontotemporal dementia, and in models of brain injury and stroke. In multiple sclerosis, elevations in CSF OPN are associated with worse disease outcomes and may be related to the ability of the cytokine to promote the survival of myelin reactive T cells (Hur et al. 2006; Vogt et al. 2004). In contrast, OPN perhaps acting through similar prosurvival pathways promotes wound healing and repair after ischemic injury (Chen et al. 2011; van Velthoven et al. 2011).

Our data provide support for the indirect pathway leading to neuronal damage and dysfunction in HIV-associated infection of the CNS. The trafficking of HIV-infected and activated macrophages, which are expressing OPN at a high level, suggests that the elevation of this cytokine in the brain would be an early event. Indeed, we found that HIV-infected individuals (without cognitive impairment) had higher levels of OPN than uninfected controls and those with MS (Brown et al. 2011). Inflammation in the brain is required for repair and normal homeostatic clearance activity. However, when inflammation is sustained over a long period of time, and at excessive levels, over activation of immune and CNS cells occur that leads, by mechanisms that remain to be fully elucidated, to functional dysregulation in which biological processes fail to behave in a normal fashion leading to cellular degeneration and death. Indeed, microglia play key roles in regulating neuronal cell death, neurogenesis, and synaptic interactions. Crosstalk through fractalkine receptor- and CD200 receptor-ligand interactions between neurons and microglia serve to regulate the inflammatory response and functional activity of these cells (Katsumoto et al. 2014).

Western blot analyses on brain cell lysates and ELISA measurements on CSF on samples from individuals with HAND revealed significant elevation in OPN that trended with the increase in cognitive impairment (Brown et al. 2011). This current study now demonstrates that the source of OPN is multifactorial coming from astrocytes, resident macrophage/microglia, and neurons. This finding expands our thinking about the possible autocrine and paracrine feedback mechanisms that can operate in the brain parenchyma to sustain OPN levels at this site. Moreover, astrocytes, microglia, and neurons all express the receptors for OPN, which include specific integrins and variants of CD44 (Ailane et al. 2013; Akiyama et al. 1993). OPN is a proinflammatory cytokine of the innate immune system that primes several downstream factors in the inflammatory cascade. Studies investigating the impact of OPN on the function of cells of the CNS in the context of HIV infection will allow us to discern whether downregulation of OPN would provide any therapeutic benefit.

## Materials and methods

### Patient samples and selection criteria

The study protocol was approved by the Johns Hopkins Institutional Review Board. Tissue sections prepared from the occipital lobe were obtained from the National NeuroAIDS Tissue Consortium (NNTC). This region of the brain has been previously shown to have abundant HIV infection and pathology. Cytomegalovirus encephalitis, toxoplasmosis (active and healed), aseptic leptomeningitis, bacterial leptomeningitis, lymphoma, contusions, focal infarcts, anoxic/ischemic damage, and tuberculosis were all excluded. In addition, for all past and current substance-induced major depressive disorder, cannabis, cocaine, opiate, and methadone use were excluded. Toxicology reports were examined and drug abuse and dependence were also excluded. All races as well as medically prescribed drugs were allowed. The revised American Academy of Neurology criteria were used to classify individuals into three groups based on cognitive function: asymptomatic neurocognitive impairment (ANI), HIV-associated mild neurocognitive disorder (MND), and HIV-associated dementia (HAD) (Antinori et al. 2007). Query of the NNTC database suggested that neurocognitive diagnosis consistent at the last two visits prior to death and the combination of MND and HAD subjects would yield the required number of cases. There were four groups examined: (1) HIV-uninfected normal controls (normal, *n* = 9); (2) neurocognitive disorder other, amyotrophic lateral sclerosis (ALS, *n* = 5); (3) HIV-infected asymptomatic neurocognitive impairment (ANI, *n* = 5); and (4) HIV-infected minor neurocognitive disorder/HIV-associated dementia (MND/HAD, *n* = 9). Case number 10 was included in the MND/HAD group based on the detection of HIV encephalitis (HIVE) on neuropathological examination.

### Immunohistochemical staining

Paraffin-embedded sections were heated for 10 min at 60 °C, then immersed in histoclear (Electron Microscopy Sciences) twice for 10 min each, followed by a 100, 95, and 70 % ethanol gradient for a total of 10 min each. Slides were rinsed in 1× Tris-buffered saline (TBS) buffer (20 mM Tris, 13.8 mM NaCl, pH 7.4) and then treated with proteinase K solution (IHC World) for 20 min at 37 °C in a humidified chamber. Depending on the antibody, slides were immersed in antigen retrieval buffer (10 mM Tris, 1 mM EDTA, 0.05 % Tween-20, pH 9.0) or citric buffer (10 mM sodium citrate, 0.05 % Tween-20, pH 6.0) and placed in a steamer for 45 min. Slides were incubated for 1 h in 10 % goat serum/TBS followed by incubation with 1:100 dilution of rabbit anti-NeuN antibody (ABN78, Millipore), rabbit anti-GFAP (AB5804, Millipore), 1:50 rabbit anti-AIF-1 (HPA049234, SIGMA-Aldrich), and 1:50 rabbit anti-CD68 (bs-1432R, Bioss) at 4 °C overnight. The slides were rinsed thoroughly in 1× TBS followed by incubation in 1:500 dilution of goat anti-rabbit-alkaline phosphatase antibody (7054, Cell Signaling) at room temperature for 1 h. Slides were rinsed in 1× TBS and developed with Permanent Fast-Red Quanto as directed by the manufacturer (Thermo Scientific) for 10–15 min at room temperature. The slides were rinsed in 1× TBS and treated with 3 % hydrogen peroxide for 5–10 min and rinsed then incubated with 1:100 of mouse anti-OPN (MAB194P, Maine Biotechnology) for 1 h at room temp. Slides were rinsed in 1× TBS then incubated with 1:1500 dilution of anti-mouse HRP (7076S, Cell Signaling) for 1 h at room temperature. Slides were developed with DAB peroxidase substrate kit (Vector) for 2 min, dehydrated, and mounted in Cytoseal 60 (Thermo Scientific).

### Data and statistical analyses

Ten or twenty images from all four quadrants of the slides were taken at ×20 (Zeiss Axio Observer A1 inverted microscope). The total area quantified was kept constant for each section analyzed. Adjustment of the image brightness, contrast, and sharpness was performed with Adobe Photoshop 5.5 using the same settings for each image. The images were saved as a new jpg file at a resolution of 300 dpi. The images were then opened in ImageJ and the colors split (Fig. 5) and pixel intensity quantified using the threshold function to select the outline of the cells of interest and measurement function to determine the area fraction of intensity. The total area examined for each sample remained constant. The area fractions determined from ImageJ were imported into GraphPad Prism 6 and analyzed by one-way ANOVA and Tukey’s correction for multiple comparisons with significance of *p* < 0.05.
